# Discovery and pharmacological characterization of a new class of prolyl-tRNA synthetase inhibitor for anti-fibrosis therapy

**DOI:** 10.1371/journal.pone.0186587

**Published:** 2017-10-24

**Authors:** Akira Shibata, Masako Kuno, Ryutaro Adachi, Yosuke Sato, Harumi Hattori, Atsushi Matsuda, Yuumi Okuzono, Keiko Igaki, Yusuke Tominari, Terufumi Takagi, Masato Yabuki, Masanori Okaniwa

**Affiliations:** 1 Immunology Unit, Pharmaceutical Research Division, Takeda Pharmaceutical Company Limited, Fujisawa, Kanagawa, Japan; 2 Biomolecular Research Laboratories, Pharmaceutical Research Division, Takeda Pharmaceutical Company Limited, Fujisawa, Kanagawa, Japan; 3 Medicinal Chemistry Research Laboratories, Pharmaceutical Research Division, Takeda Pharmaceutical Company Limited, Takeda Pharmaceutical Company Limited, Fujisawa, Kanagawa, Japan; Keio University, JAPAN

## Abstract

Scleroderma has clinical characteristics including skin and other tissue fibrosis, but there is an unmet need for anti-fibrotic therapy. Halofuginone (HF) is a well-known anti-fibrosis agent in preclinical and clinical studies which exerts its effect via inhibition of TGF-β/Smad3 signaling pathway. Recently, prolyl-tRNA synthetase (PRS) was elucidated as a target protein for HF that binds to the proline binding site of the catalytic domain of PRS. Here, we characterized a new class of PRS inhibitor (T-3833261) that is carefully designed in a way that binds to the ATP site of the catalytic domain and does not disrupt binding of proline. The anti-fibrotic activity and the mechanism of action for T-3833261 on TGF-β-induced fibrotic assay were compared with those of HF in primary human skin fibroblast. We evaluated *in vivo* effect of topical application of T-3833261 and HF on TGF-β-induced fibrotic genes expression in mice. We found that T-3833261 suppressed TGF-β-induced α-smooth muscle actin (α-SMA) and type I collagen α1 (COL1A1) expression through the Smad3 axis in a similar fashion to HF. *In vivo* topical application of T-3833261 reduced the increase of fibrotic genes expression such as α-Sma, Col1a1 and Col1a2 by TGF-β intradermal injection to the ear of a mouse. We revealed that T-3833261 is more effective than HF under the conditions of high proline concentration, as reported in fibrotic tissues. These results suggest the potential of ATP competitive PRS inhibitors for the treatment of fibrotic diseases such as scleroderma.

## Introduction

Scleroderma is a multisystem autoimmune disorder characterized by initial vascular injuries and resultant fibrosis of the skin and certain internal organs [1, 2]. Although the pathogenesis of scleroderma remains unknown, it has been observed that during the course of the disease, there is an excessive accumulation of extracellular matrix (ECM) components in the skin and other tissues [3]. The accumulation of collagen type I in scleroderma patients is mediated by activated skin fibroblasts, which leads various fibrotic phenotypes containing collagen type I proteins production [4]. While various cytokines and growth factors are considered to contribute to skin fibroblast activation in scleroderma, transforming growth factor-β (TGF-β) plays an important role in the fibrotic reaction of scleroderma pathology [5, 6]. The monoclonal antibody of TGF-β, Fresolimumab, has been recently shown to improve the modified Rodnan skin score (mRSS) in scleroderma patients in a Phase-2 clinical study [7]. However, until now, no drug has been approved as an anti-fibrotic capable of preventing progression or recovery from existing fibrosis.

Halofuginone (HF), a plant alkaloid derivative, is a well-known inhibitor of collagen type I production via inhibition of the TGF-β-induced Smad3 pathway [8, 9]. Previously, topical treatment of HF to chronic graft versus host disease and scleroderma patients caused a transient attenuation of collagen I gene expression and improvement of skin fibrotic score, leading to human clinical efficacy [10, 11]. Recently HF has been shown to bind glutamyl-prolyl-tRNA synthetase inhibiting prolyl-tRNA synthetase (PRS) activity [12]. HF has been reported as a PRS inhibitor that increases phosphorylation of general control nonderepressible 2 (GCN2) and leads to activating transcription factor 4 (ATF4) and DNA Damage Inducible Transcript 3 (DDIT3) expression as an amino acid starvation response [12]. Interestingly, PRS inhibition by HF is attenuated by the addition of exogenous proline because HF competitively binds to the proline binding pocket of the catalytic site of PRS [12]. This nature of HF was also reported as a cause of phenotypic drug resistance through the accumulation of proline, in an article that describes the application of HF as a Plasmodium falciparum PRS inhibitor for the anti-malarial agent [13]. In fibrotic tissue, the concentration of proline is higher than that of non-fibrotic tissues [14]. This suggests that accumulated proline in fibrotic tissues would attenuate the anti-fibrotic effect of HF. Based on this evidence, we hypothesized that the PRS inhibitor that does not compete with proline would overcome this issue.

**Fig 1:**
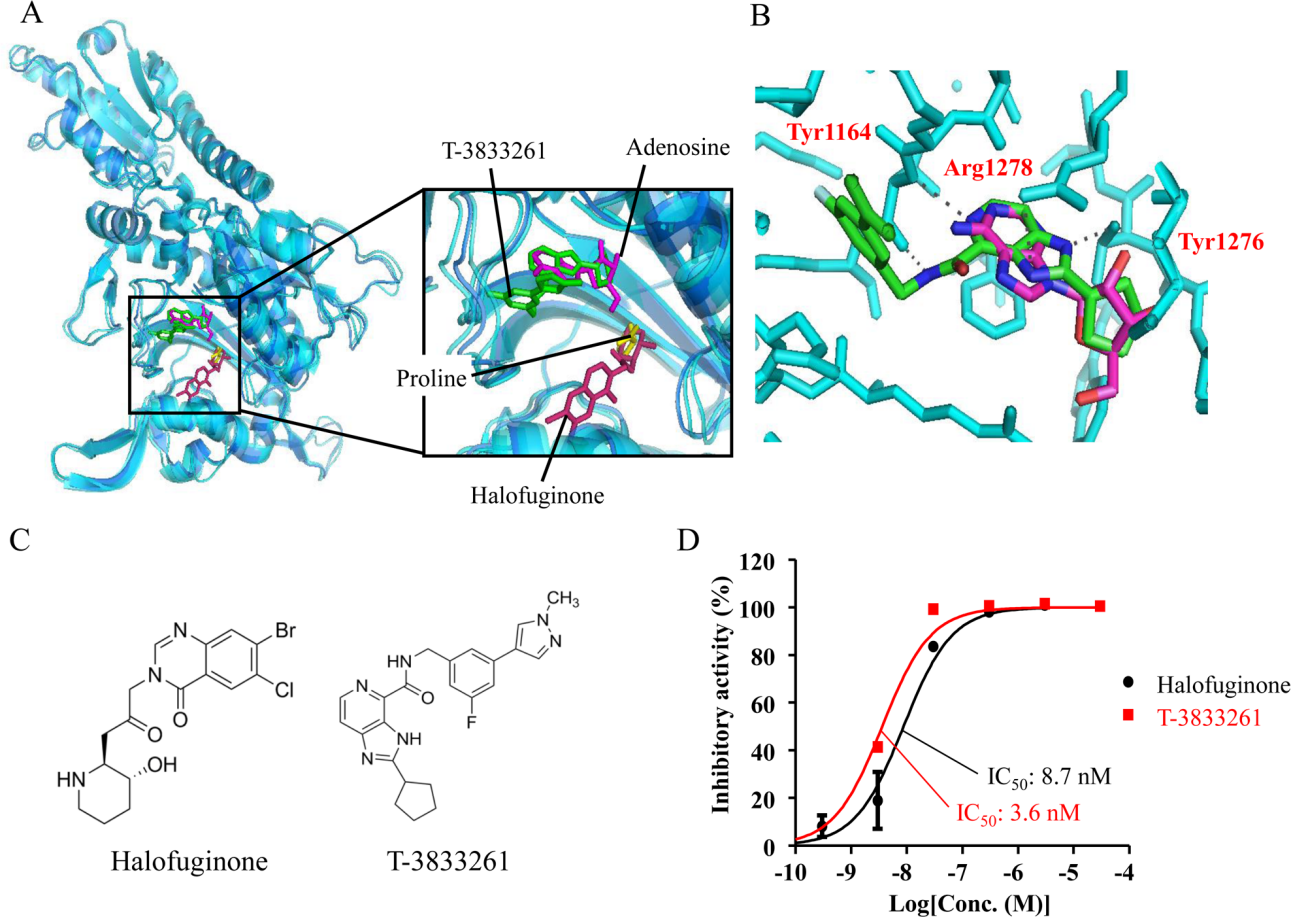
T-3833261 is a potent ATP competitive PRS inhibitor. (A) Molecular model constructed by available PRS crystal structures bearing adenosine and proline (PDB: 4K87) and Halofuginone (PDB: 4K88), (B) Binding mode of T-3833261 in the ATP binding site, (C) Chemical structure, (D) Biochemical activity of compound in PRS inhibition measured by an ATP/PPi exchange method. Results performed in duplicate are shown as the mean ± SD. The inhibitory activities are expressed as the percentage of vehicle-treated control.

To achieve this targeted profile, an ATP binding site in proximity to the proline binding site in the catalytic domain of PRS was highlighted. We discovered a new ATP competitive PRS inhibitor with different inhibitory modes from HF by using an established screening system [15]. By using cocrystal structures of PRS protein bearing either HF or our lead compound, potent PRS inhibitor T-3833261 was designed in a way that binds to the ATP site and does not bind to the proline binding site (Fig 1A). Recently, our lead molecules were reported to exert potent amino acid starvation responses with GCN2-ATF4 pathway activation and showed selective cell death against cancer cells, such as SK-MEL-2, that are sensitive to amino acid deprivation [16]. In this report, the anti-fibrotic activity and the mechanism of action for new ATP-competitive PRS inhibitor T-3833261 on TGF-β-induced fibrotic assay were compared with those of HF *in vitro*. In addition, we evaluated *in vivo* effect of topical application of T-3833261 and HF on TGF-β-induced fibrotic genes expression in mice. Finally, we characterized the difference between two PRS inhibitors with distinct binding modes under high proline concentration conditions, which is frequently observed in fibrotic tissues.

## Materials and methods

### Reagents

HF was purchased from Wako Pure Chemical Industries (Osaka, Japan). T-3833261, 2-Cyclopentyl-*N*-(3-fluoro-5-(1-methyl-1*H*-pyrazol-4-yl)benzyl)-3*H*-imidazo[4,5-*c*]pyridine-4-carboxamide was synthesized at the Shonan Research Center in Takeda Pharmaceutical Company Limited. Compounds were dissolved in dimethyl-sulfoxide (DMSO, Wako Pure Chemical Industries, Japan) at a concentration of 10 mM. The final concentration of DMSO in the medium was less than 0.1% (v/v), which did not affect cell viability. A medium with DMSO alone was similarly prepared and used as vehicle control (0.1% DMSO). All other chemicals used were of highest grade commercially available.

### Protein preparation

The expression plasmid of human PRS (residues 998–1512, Genbank Accession No. NM_152621) was constructed in a pET21a vector (Novagen, San Diego, CA, USA) to express in *E*.*coli* BL21 (DE3) (Nippongene, Tokyo, Japan) as a fusion protein with His-Avi-tag at N-terminus. After culture, the cells were homogenized in a lysis buffer containing 50 mM Tris-HCl (pH 8.0 at 25°C), 150 mM NaCl, 5 U/mL Benzonase, 20 mM imidazole, and 1 mM DTT. Cell homogenates were centrifuged, and the supernatant was applied to NiNTA purification, followed by gel filtration with Superdex200. Proteins were stored in a buffer containing 50 mM Tris-HCl (pH 8.0 at 25°C), 150 mM NaCl, 10% glycerol, and 1 mM DTT at -80°C. The protein concentration was determined with the BCA Protein Assay Kit (Pierce Biotechnology, Inc., Rockford, IL, USA) according to the instruction manual.

### ATP/PPi exchange assay

The test compounds were dissolved in 5 μL of an assay buffer [50 mM Tris-HCl (pH 7.5), 20 mM KCl, 1 mM DTT, and 0.01% Tween 20] and incubated with 5 μL of 40 nM PRS enzyme dissolved in the assay buffer supplemented with 40 mM MgCl_2_ for 60 minutes. The reaction was started by the addition of 10 μL of a substrate solution containing 300 μM ATP, 160 μM L-Proline, 400 μM pyrophosphate, and trace amount of ^32^P-labelled pyrophosphate (PerkinElmer, Norwalk, CT, USA). After incubation at room temperature for 30 minutes, the reaction was stopped with 50 μL of a stop/wash buffer (1 M HCl and 200 mM sodium pyrophosphate). The reaction solution was transferred to a filter plate (Merck Millipore, Bedford, MA, USA) which was dispensed with 200 μL of a charcoal solution (10% charcoal (w/v) in 0.5% HCl). The filter plate was washed with the wash buffer for five times. The radiolabeled product was eluted with an elution buffer (2 M ammonia in 60% ethanol (v/v)) to a 96 well OptiPlate (PerkinElmer) and added with microscinti 20 (PerkinElmer). Radioactivities were measured using the TopCount detector (PerkinElmer).

The inhibitory activity was calculated as follows: % inhibition = (A—B) / (A—C) × 100, where, A, B, and C are signals with vehicle, test sample, and without reaction, respectively. The dose–response data were then fitted to a four-parameter logistic curve using GraphPad Prism (GraphPad Software, San Diego, CA, USA) to determine IC_50_ values for test compounds.

### Docking model prediction

Compound T-3833261 was docked into the crystal structure of PRS protein using GOLD (version 5.4.1, the Cambridge Crystallographic Data Centre) with the standard default settings. The initial structure of the compound-complexed PRS model was energy-minimized using the MMFF94s force field in MOE (version 2015.10, Chemical Computing Group) to obtain the final docking models. During the minimization procedure, the following conditions were adopted. The dielectric constant was set to 4r, where r is the distance between the two interacting atoms. The residues, which are 8 Å away from each compound, were fixed. A harmonic force constraint against the initial atomic positions of the backbone was added, using 0.3 Å as a force constant. The atomic charges for the protein and the compounds were set according to the AMBER99 force field and the AM1-BCC method, respectively.

### Cell culture

Primary human skin fibroblasts (NHDF-Ad) were obtained from Lonza (Basel, Switzerland). Cells were cultured and maintained in growth medium: Fibroblast Basal Medium (FBM) containing human fibroblast growth factor-B, insulin, fetal bovine serum (FBS) and gentamicin/amphotericin-B (Lonza). Cells were maintained in a humidified incubator at 37°C with 5% CO_2_ and used for experiments between passages 5 to 8.

### α-smooth muscle actin (α-SMA) and pro-collagen protein expression

Protocol 1: Cells were allowed to adhere overnight before being replaced with serum-free FBM for 24 h. Serum-starved cells were first pretreated with samples in DMSO at concentrations between 1 and 300 nM for 30 min and then stimulated with 1 ng/mL recombinant human TGF-β (R&D Systems, Minneapolis, MN, USA) for 24 h.

Protocol 2: Cells were allowed to adhere overnight before being replaced with serum-free FBM for 24 h. Cells were then treated with TGF-β (1 ng/mL) for 48 h to induce differentiation to myofibroblast with expressing α-SMA protein. The medium was then removed, and the cells were subsequently treated with or without samples (1–300 nM) in serum-free FBM for an additional 48 h.

Cell lysates were prepared with cell lysis buffer (Cell Signaling Technology, Beverly, MA, USA) and subjected to α-SMA ELISA system (Abnova, Taipei, Taiwan) and Pro-type I Collagen α1 (pro-COL1A1) SimpleStep ELISA Kit (Abcam, Cambridge, UK, USA) according to the manufacturer's protocol.

### RNA preparation *in vitro*

Cells were allowed to adhere overnight before being replaced with serum-free FBM for 24 h. Serum-starved cells were first pretreated with samples in DMSO at concentrations between 1 and 300 nM for 30 min and then stimulated with 1 ng/mL TGF-β for 24 h. Then total RNA was collected from the cells by RNeasy 96 Kit (Qiagen, Valencia, CA, USA) and DNaseI (Qiagen) according to the manufacturer's protocol.

### Smad3 activation in western blotting

Cells were allowed to adhere overnight before being replaced with serum-free FBM for 24 h. Serum-starved cells were first pretreated with samples in DMSO at 30 and 300 nM for 24 h and then stimulated with 1 ng/mL TGF-β for 30 min. Cell lysates were prepared with lysis buffer and cellular proteins (11 μg/well) were separated by sodium dodecyl sulfate-polyacrylamide gel electrophoresis (4–20% Criterion TGX Precast Gel; Bio-Rad, Ontario, CA, USA). Protein bands were then transferred to polyvinylidene fluoride membranes (Trans-Blot Turbo Midi, Bio-Rad). Following blocking, the membranes were incubated with primary antibodies targeted against phospho-Smad3 (Cell Signaling Technology), Smad3 (Cell Signaling Technology) and β-actin (Cell Signaling Technology) followed by horseradish peroxidase-conjugated secondary antibody (Cell Signaling Technology). ECL Prime Western Blotting Detection Reagent (GE Healthcare Bio-Sciences, Little Chalfont, England) and LAS-3000 image analyzer (Fuji-film, Tokyo, Japan) were used for detection. Western blot band intensities were calculated by densitometric analyses with National Institutes of Health image software, Image J. Representative pictures are shown with quantified densitometric data.

### Animal ethics and procedures

Animal studies are reported in compliance with the ARRIVE guidelines [17, 18]. Efforts were made to minimize animal suffering as much as possible during experiments. The experimenters were blinded to the treatments given to the animals and to the biochemical and histological analyses and the data analyses. Eight-week-old C3H/He male mice (weighing 23~28 g, Japan SLC, Inc., Hamamatsu, Japan) were used for all the *in vivo* experiments. Mouse experiments were done following guidelines and a protocol was performed in accordance with the standards of humane care. The treatment of research animal was approved by the Institutional Animal Care and Use Committee of Pharmaceutical Research Division, Takeda Pharmaceutical Company Limited (Approval No. 11084). These animals were maintained on a 12/12 h light/dark cycle with constant temperature (23 ± 2°C) under specific pathogen-free conditions. Standard chow diet and water were available ad libitum.

### Fibrotic gene expression *in vivo*

After acclimatization for 1 weeks, animals were randomly divided according to body weight and treated for 3 to 5 consecutive days as follows. The ear and dorsal skin of mouse were intradermally injected with 20 μL (100 μL, dorsal skin) PBS or 500 ng TGF-β in PBS under anaesthesia with 3–4% sevoflurane. Injections were continued in this manner daily for 3 days (days 0, 1 and 2) or 5 days (days 0, 1, 2, 3 and 4). Treatment protocol included the daily topical application of T-3833261 or HF cream on the ear (40 μL/ear) 1 h before injections into the ear. A vehicle without compounds was applied to control mice. The topical material contained sorbitan sesquioleate (50 g), lanolin (50 g), liquid paraffin (250 g), propylene glycol (50 g), 90% lactic acid (1.5 g), T-3833261 (0.1, 0.3 and 1 g) or HF (0.1 g), and distilled water as the rest material. Topical application of compound was continued for 7 days. Mice were euthanized by cervical dislocation under sevoflurane anesthesia at 3 or 5 days after last TGF-β injection and the ear surrounding the TGF-β injection site was harvested with 8 μm biopsy punch under anesthesia. A portion of the ear samples was treated with RNA-later (Ambion, Austin, TX, USA) for real-time RT-PCR.

### Real-time quantitative RT-PCR

Total RNA was isolated from *in vivo* samples using ISOGEN II (Nippongene) according to the manufacturer's instructions. *In vitro* RNA samples were prepared as mentioned above. High Capacity cDNA Reverse Transcription Kit (Life Technologies, Carlsbad, CA, USA) was used for cDNA synthesis. Real-time quantitative RT-PCR reactions were performed as a TaqMan method on a ViiA 7 real-Time PCR System (Life Technologies), using TaqMan Fast Advanced Master Mix (Lifer Technologies) with specific primers on TaqMan Gene Expression Assays (Life Technologies) according to the manufacturer's manual. FAM-probed primers were used: COL1A1, Hs00164004_m1; α-SMA/ACTA2, Hs00426835_g1; fibroblast growth factor 2 (FGF2), Hs00266645_m1; Smad specific E3 ubiquitin protein ligase 2 (SMURF2), Hs00224203_m1; DDIT3, Hs00358796_g1; and glyceraldehyde 3-phosphate dehydrogenase (GAPDH), 4326317E for human *in vitro* cell assay. FAM-probed and TAMRA-quenched primers (Sigma-Aldrich Japan, Tokyo, Japan) were used: Col1a1, NM_007742; type I collagen alpha 2 (Col1a2), NM_007743; α-Sma/Acta2, NM_007392; and Gapdh, NM_008084 for mouse *in vivo* assay (S1 Table). The relative mRNA level of the target genes was calculated by the comparative threshold cycle (Ct) method using GAPDH (Gapdh) as an internal control for normalization. The fold change in the expression of each target gene was calculated by the following formula: relative quantification (RQ) = 2^-ΔΔCt^.

### Proline addition assay

To evaluate the effect of proline addition on anti-fibrosis by each compound, the cells were allowed to adhere overnight before being replaced with serum-free FBM for 24 h. Serum-starved cells were first pretreated with samples at concentrations between 1 and 300 nM and 0.05–1.0 mM L-proline (SIGMA, St. Louis, MO, USA) for 30 min and then stimulated with 1 ng/mL TGF-β for 24 h. Then total RNA was collected from the cells by RNeasy 96 Kit. Then real-time quantitative RT-PCR analysis was conducted as mentioned above.

### Transcriptome analysis

The Ion AmpliSeq Transcriptome Human Gene Expression Kit (ThermoFisher, Waltham, MA, USA) enables the simultaneous measurement of the expression levels of over 20,000 human genes in a single assay. AmpliSeq human-transcriptome libraries were constructed and sequenced by using the Ion Proton platform, according to the manufacturer’s instructions. Briefly, total RNA was extracted from human skin fibroblast treated with compounds for 24 h using the RNeasy Mini Kit. Reverse transcription was performed on 10 ng of the prepared total RNA samples using the AmpliSeq Whole Transcriptome primers with the included SuperScript® VILO^TM^ cDNA Synthesis kit (Life Technologies). Amplicons were ligated to adapters, and the resulting libraries were purified using Agencourt AMPure XP reagents (Beckman Coulter, Brea, CA, USA) and diluted to the concentration of 75 pM. Templated libraries were sequenced with the Ion Proton sequencer (ThermoFisher).

### Gene signature analysis

AmpliSeq sequencing data were analyzed using the Ion Torrent Mapping Alignment Program. Identification of differentially expressed genes (DEGs) and comparative gene signature analysis were done in NextBio (http://nextbio.com). Gene expression data was taken by using our ATP-competitive PRS inhibitor analog (IC_50_: < 3.0 nM). Data from PRS inhibitor analog-treated human skin fibroblasts were compared to data from HF-exposed human skin fibroblasts to assess similarities of the effects of both compounds on gene expression profiles. The common gene signature from PRS inhibitor analog and HF exposed human skin fibroblasts were compared to curated datasets to evaluate the correlation between PRS inhibitors common signature and scleroderma disease signature (GE4385). We used the NextBio Application for the identification of enriched functional categories among DEGs in between PRS inhibitors common expression and GE4385 of scleroderma expression. We assessed enrichment of biological processes in gene ontology (GO) and showed the top 10 correlated processes.

### Statistics

Statistical analysis was performed using SAS System for Windows (Release 9.3, SAS Institute). The significant differences between groups of normally distributed data was determined by Student’s t-test (comparison between two groups). Welch's t-test was used when the data has unequal variances. Values of p<0.05 were considered statistically significant. Differences between the vehicle-treated control group and compounds-treated groups were analyzed for homogeneity of variance using Bartlett's test. Parametric data were analyzed by one-tailed Williams' test. Nonparametric data were analyzed using Shirley-Williams' test. One-way ANOVA with post hoc Dunnett’s test (comparison between multiple groups) for in *vivo* experiment.

## Results

### PRS inhibitors exhibited significant inhibitory activity to PRS enzyme

We synthesized the novel fused pyridine derivative T-3833261 based on structure-based drug design using the X-ray crystal structure of PRS protein bearing our lead molecule (Fig 1A and 1C) and found that this compound showed comparable PRS enzymatic inhibition (PRS IC_50_: 3.6 nM) to HF (PRS IC_50_: 8.7 nM) in an ATP/PPi exchange assay utilizing PRS (Fig 1D). In our docking model, T-3833261 is accommodated in the ATP binding site in proximity to the proline binding site that is occupied by proline or HF (Fig 1A). The carbamoyl pyridine moiety of T-3833261 occupied an adenine position in the ATP binding site, and the NH of the carbamoyl group interacted with the carbonyl group of the Tyr1164 (Fig 1B). Nitrogen atoms of the imidazopyridine scaffold formed hydrogen bond interactions with Arg1278 and Tyr1276 residues. Moreover, the cyclopentyl group occupied a ribose position in the ATP binding site. This docking model confirmed that our molecule was accommodated in the ATP binding site and did not disrupt proline binding.

### PRS inhibitors dose-dependently decreased the TGF-β-induced α-SMA and pro-COL1A1 protein levels in human skin fibroblast

**Fig 2:**
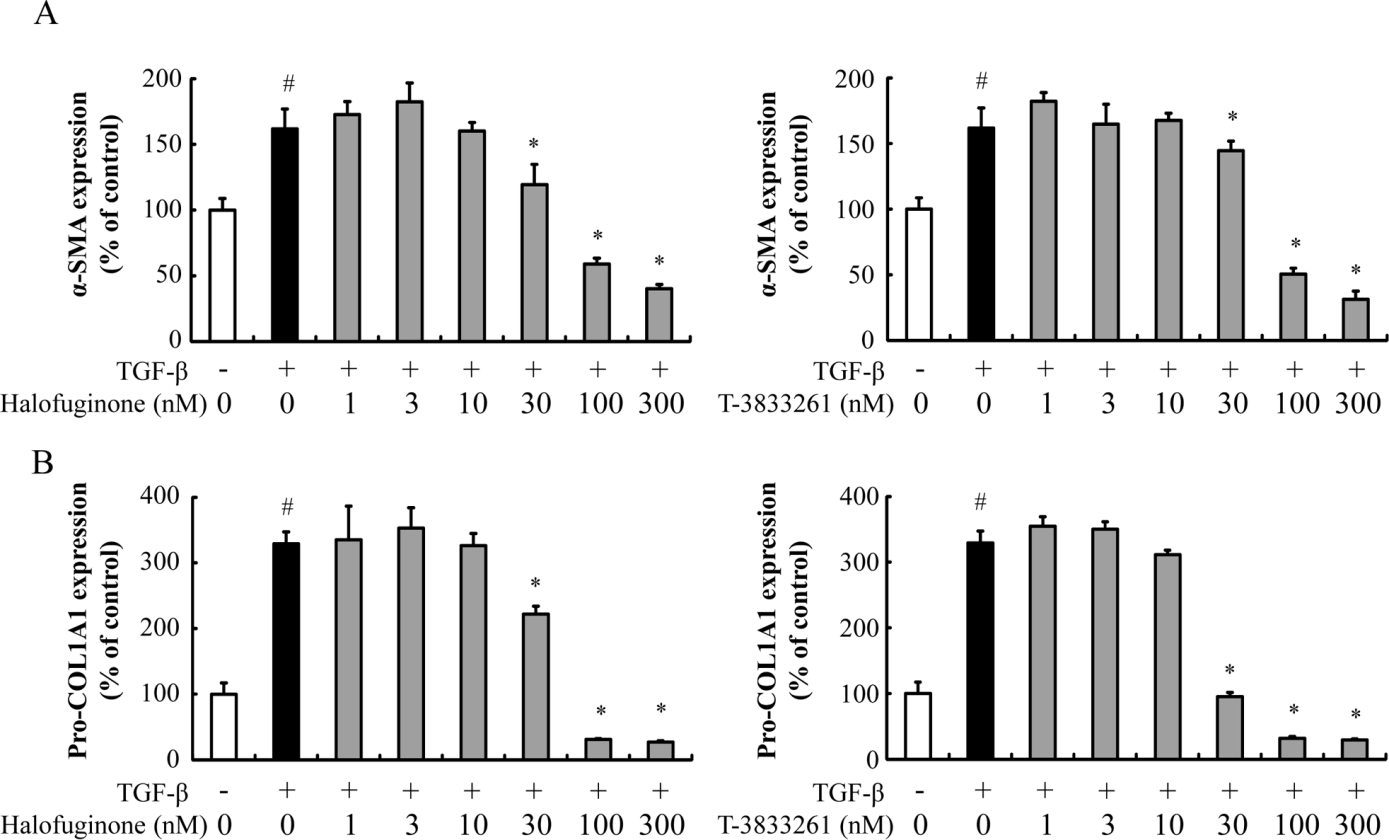
Effect of T-3833261 or Halofuginone on α-SMA and pro-COL1A1 protein content in TGF-β-treated human skin fibroblast. Skin fibroblasts were pre-treated with samples (1–300 nM) for 0.5 h, followed by stimulation with TGF-β (1 ng/mL). After incubation for 24 h, α-SMA (A) and pro-COL1A1 (B) protein levels were measured by ELISA. The expression levels are expressed as the percentage of vehicle-treated control. Values are mean ± SD (n = 4). #p<0.05 compared to vehicle-treated control, *p<0.05 compared to TGF-β-treated control. The experiment was repeated by using other fibroblast lots and similar results were obtained.

In the fibrotic process, α-SMA and type I collagen are known to increase significantly in myofibroblasts and have been commonly used as fibrotic markers. Stimulation of TGF-β induced the α-SMA protein expression, which is a fibrosis-related molecule in human skin fibroblast (Fig 2). HF and T-3833261 suppressed α-SMA protein expression in a dose-dependent manner (Fig 2A). TGF-β also induced the pro-COL1A1 protein expression in skin fibroblast. HF and T-3833261 suppressed pro-COL1A1 protein expression in a dose-dependent manner (Fig 2B). Both HF and T-3833261 reduced α-SMA and pro-COL1A1 proteins below the basal level of non-stimulated cells at a concentration of 100 nM or above. The drug concentrations we used here showed marginal effects on cellular cytotoxicity.

### PRS inhibitors reduced the Smad3 expression

**Fig 3:**
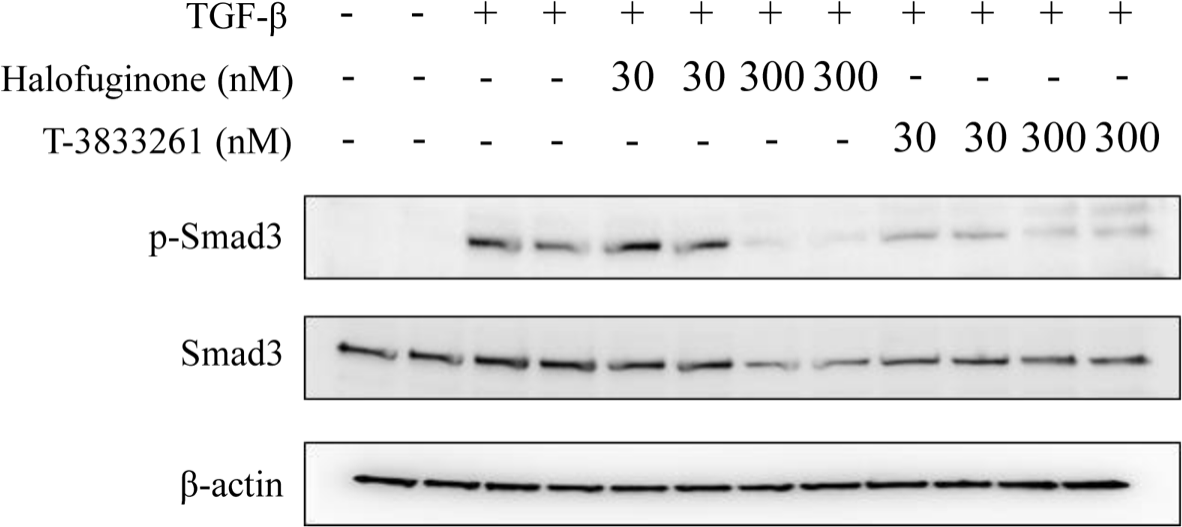
Effect of T-3833261 or Halofuginone on Smad3 protein expression in human skin fibroblasts. Representative western blots show that Halofuginone or T-3833261 reduces Smad3 and phosphorylated Smad3 (p-Smad3) protein expressions significantly. β-actin shown as simultaneous loading controls. The data of this experiments were repeated three times.

Smad3 is a key mediator of the TGF-β-dependent fibrotic response and Smad3 signaling has become a promising target for anti-fibrotic therapies. Both genetic and chemical approaches have been developed to regulate this pathway [19, 20]. A previous study demonstrated that HF inhibits TGF-β-induced expression of fibrotic markers such as α-SMA and COL1A1 via down-regulating Smad3 expression in human corneal fibroblasts [8]. To confirm whether T-3833261 has a similar effect, we tested the level of Smad3 protein after 24 h treatment with HF and T-3833261 in human skin fibroblasts (Fig 3). HF showed a decrease in the TGF-β-induced accumulation of Smad3 and phosphorylated Smad3 (p-Smad3) at 300 nM. On the other hand, T-3833261 showed a decrease in Smad3 and p-Smad3 at 30 and 300 nM, respectively.

### PRS inhibitors suppressed the expression of fibrosis-related genes in primary skin fibroblasts

**Fig 4:**
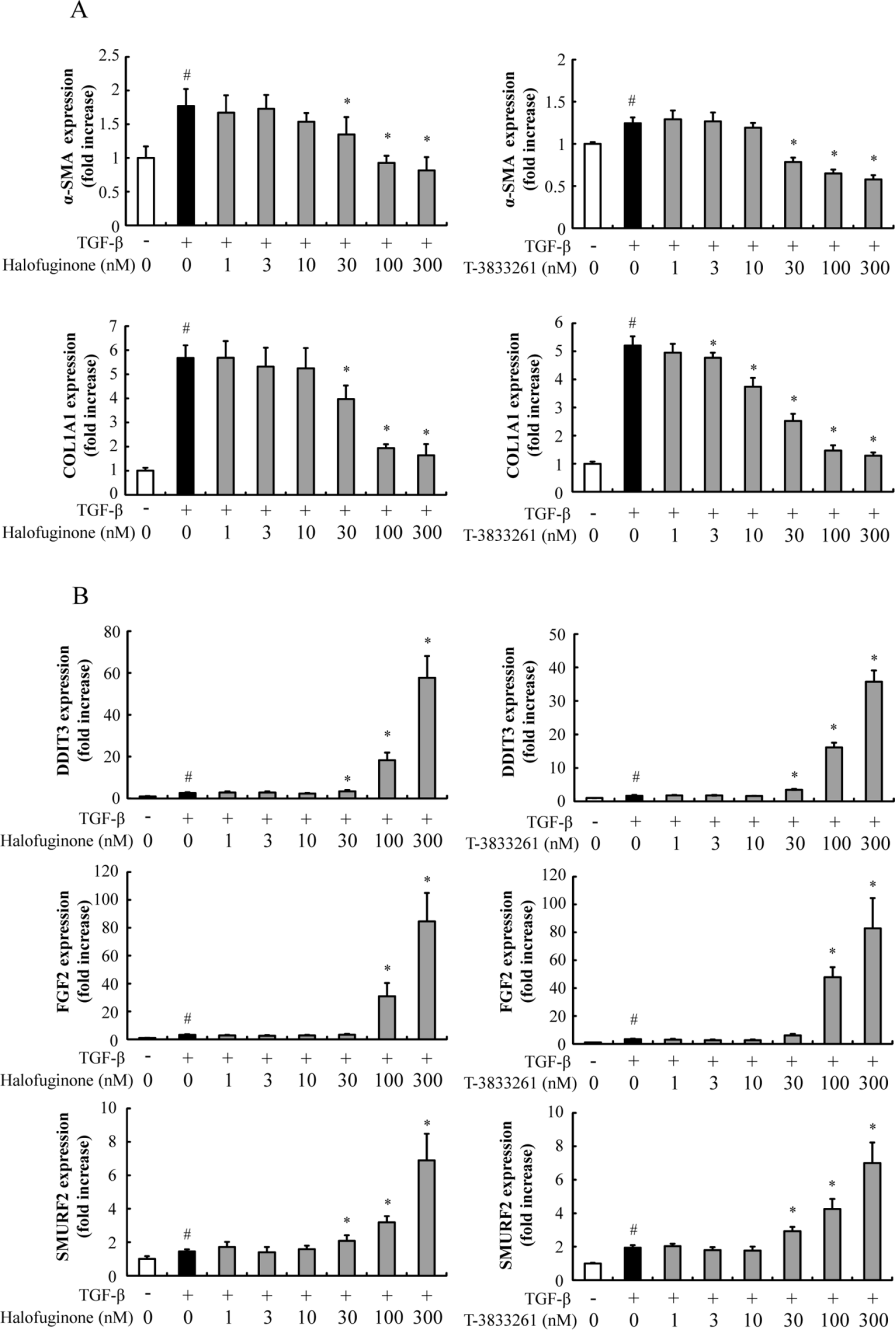
Effects of T-3833261 or Halofuginone on mRNA expression of fibrosis-related genes. Skin fibroblasts were pre-treated with samples (1–300 nM), followed by stimulation with TGF-β (1 ng/mL). After incubation for 24 h, α-SMA, COL1A1 (A), DDIT3, FGF2 and SMURF2 (B) mRNA levels were measured by real-time quantitative RT-PCR. Gene expression levels (normalized to GAPDH) are expressed as the fold change of vehicle-treated control. Values are mean ± SD (n = 6). #p<0.05 compared to vehicle-treated control, *p<0.05 compared to TGF-β-treated control. The experiment was repeated by using other fibroblast lots and similar results were obtained.

Real-time RT-PCR confirmed that TGF-β increased the expression of α-SMA and COL1A1 in primary human skin fibroblast cultures compared to vehicle treatment (Fig 4A). HF treatment suppressed TGF-β-induced expression of α-SMA. In addition COL1A1 was reduced to near the level of non-stimulated control. T-3833261 also suppressed expression of these fibrotic genes, and showed equivalent inhibitory effect compared to that of HF. HF and T-3833261 dose-dependently increased DDIT3 expression, which indicated the induction of amino acid starvation response by PRS inhibition (Fig 4B). Additionally, HF and T-3833261 dose-dependently increased SMURF2 and FGF2 expressions, which are related to the anti-fibrotic mechanism (Fig 4B).

### Consecutive TGF-β injection induced fibrotic marker expression in ear of mouse

**Fig 5:**
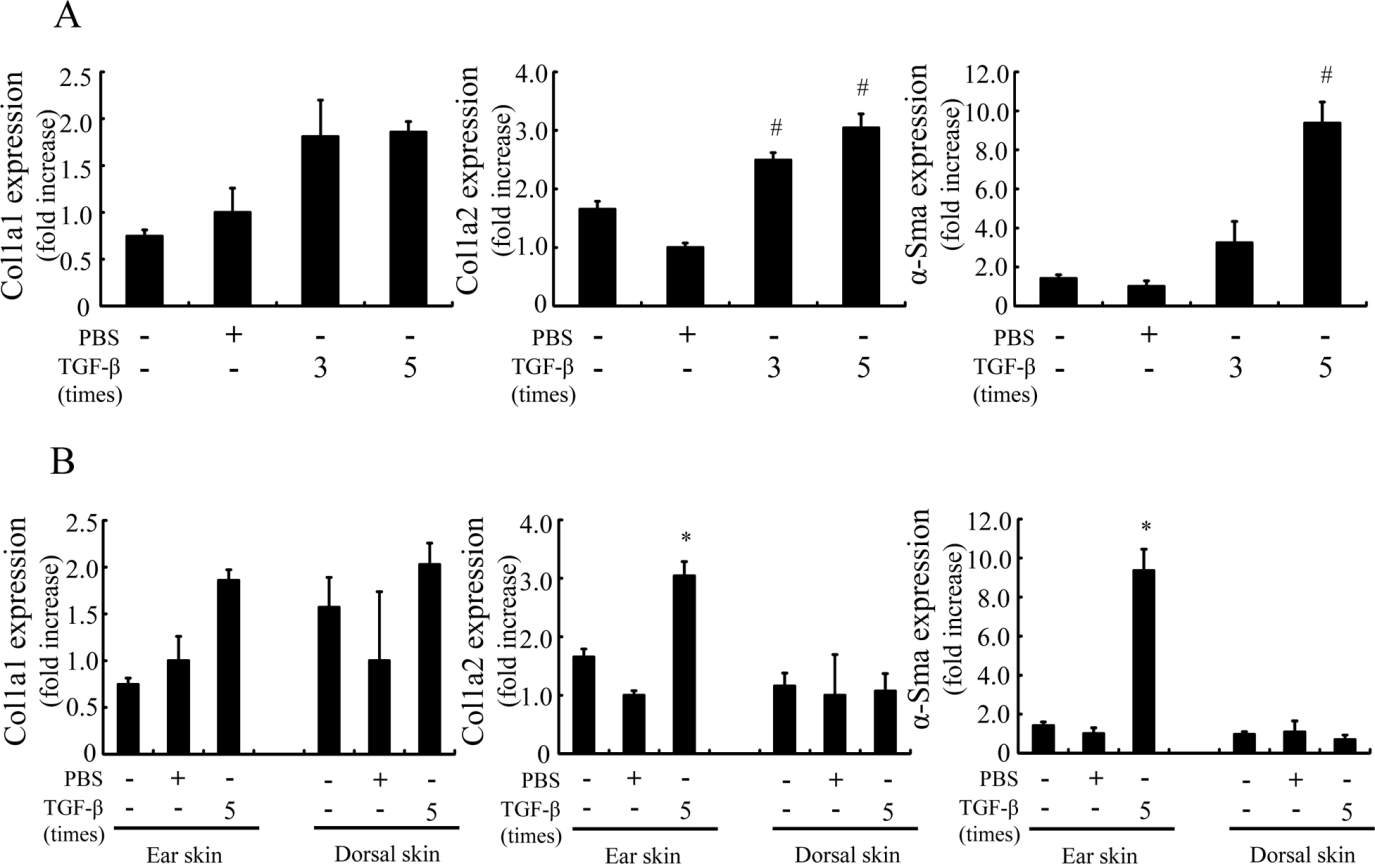
Fibrotic genes expression of ear and dorsal skin in a mouse by TGF-β injection. TGF-β (500 ng/20 μL) or vehicle was intradermally injected 3 or 5 times in the ear (A) or dorsal skin (B) of mice. mRNA expression of α-Sma, Col1a1 and Col1a2 in the ear was measured 3 or 5 days after final TGF-β administration was evaluated. Gene expression levels (normalized to Gapdh) are expressed as the fold change of vehicle-treated control. Values are mean ± SE (n = 2–5). #p<0.05 compared to PBS-injected normal mice, *p<0.05 compared to PBS-injected normal mice.

The TGF-β-treated group increased fibrosis-related genes expression such as Col1a1, Col1a2 and α-Sma in mouse ear compared to the control group. As shown in Fig 5A, five injections of TGF-β clearly induced fibrotic genes expression compared to three injections. Furthermore, since fibrotic gene expression of the ear was higher than that of dorsal skin, the ear was a more suitable site compared to the dorsal skin as a TGF-β injection site for evaluating fibrotic gene expression (Fig 5B). In the next experiment, as a stable fibrotic condition with greatest reproducibility, we adopted the TGF-β-injection to mouse ear on days 0–4 (5 times) protocol as the preferred experimental condition.

### Topical application of PRS inhibitor reduced fibrotic gene expression in TGF-β-induced mouse model

**Fig 6:**
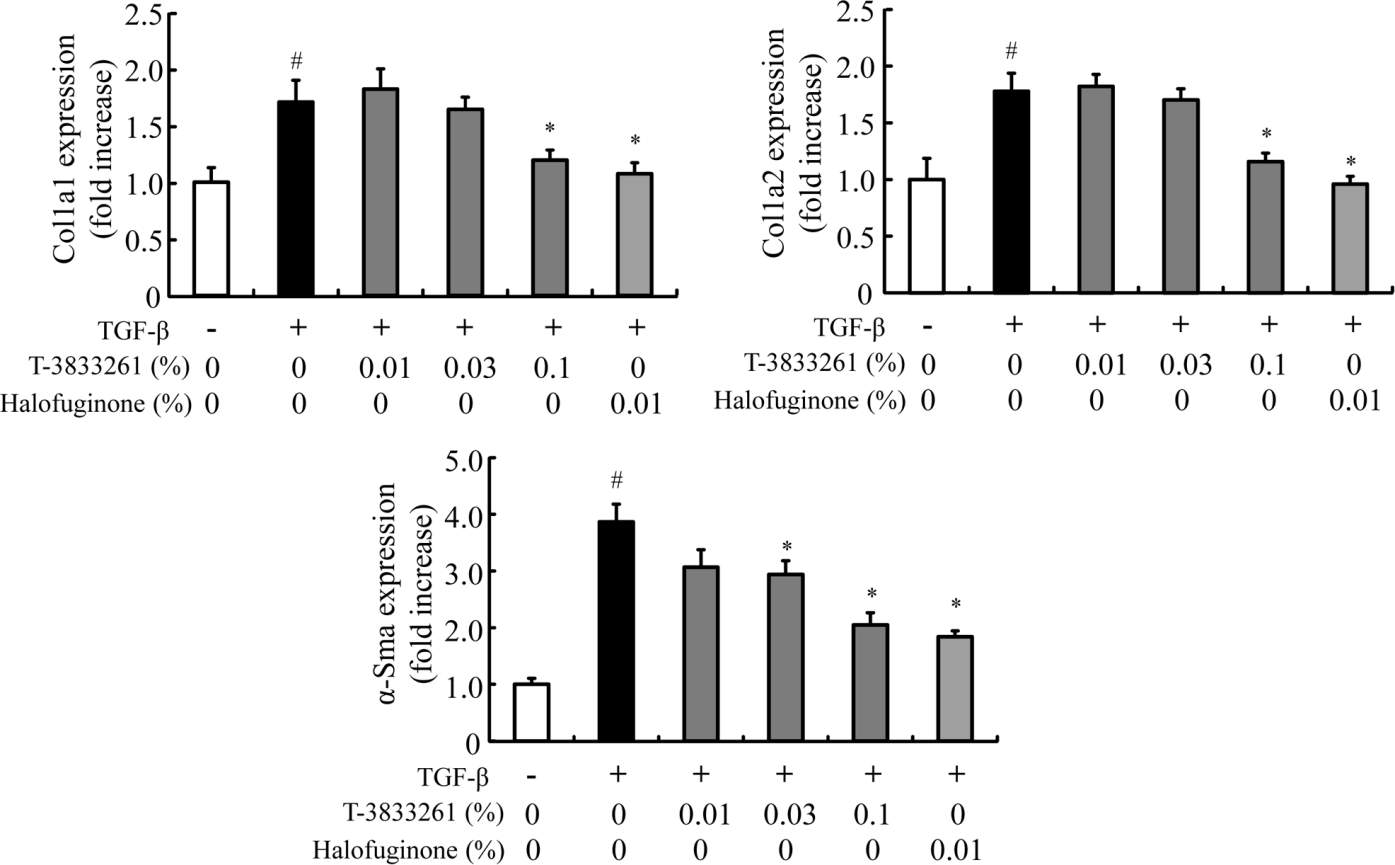
Effects of topical T-3833261 or Halofuginone on TGF-β-induced fibrotic genes expression in mouse ear skin. TGF-β (500 ng/20 μL) or vehicle was intradermally injected 5 times in the ear of mice. T-3833261, Halofuginone or vehicle was locally administered to the mice 1 h before TGF-β injection. Skin biopsies were taken from each sample treated mice. mRNA expression of α-Sma, Col1a1 and Col1a2 in the ear was measured 78 h after final TGF-β administration was evaluated. Gene expression levels (normalized to Gapdh) are expressed as the fold change of vehicle-treated control. Values are mean ± SE (n = 4–8). #p<0.05 compared to PBS-injected normal mice, *p<0.05 compared to TGF-β-injected mice.

*In vivo* anti-fibrosis activities of the compound were assessed by topical application of a cream formulation. TGF-β significantly increased expression of fibrosis-related genes such as Col1a1, Col1a2 and α-Sma in mouse ear, and topical application of HF prevented the skin fibrotic genes expression induced by TGF-β (Fig 6). Topical application of T-3833261 also reduced expression of these fibrotic genes in a dose-dependent manner, with a 0.1% formulation of T-3833261 showing comparable activity to a 0.01% HF formulation. The body weight of the mice did not change after one week of continuous application of topical HF and T-3833261 (S2 Table). These results confirmed that both T-3833261 and HF are well-tolerated and demonstrate anti-fibrotic effect in *in vivo* settings when they are administered by dermal topical application.

### Proline addition reversed the anti-fibrotic effects of HF, but not T-3833261

**Fig 7:**
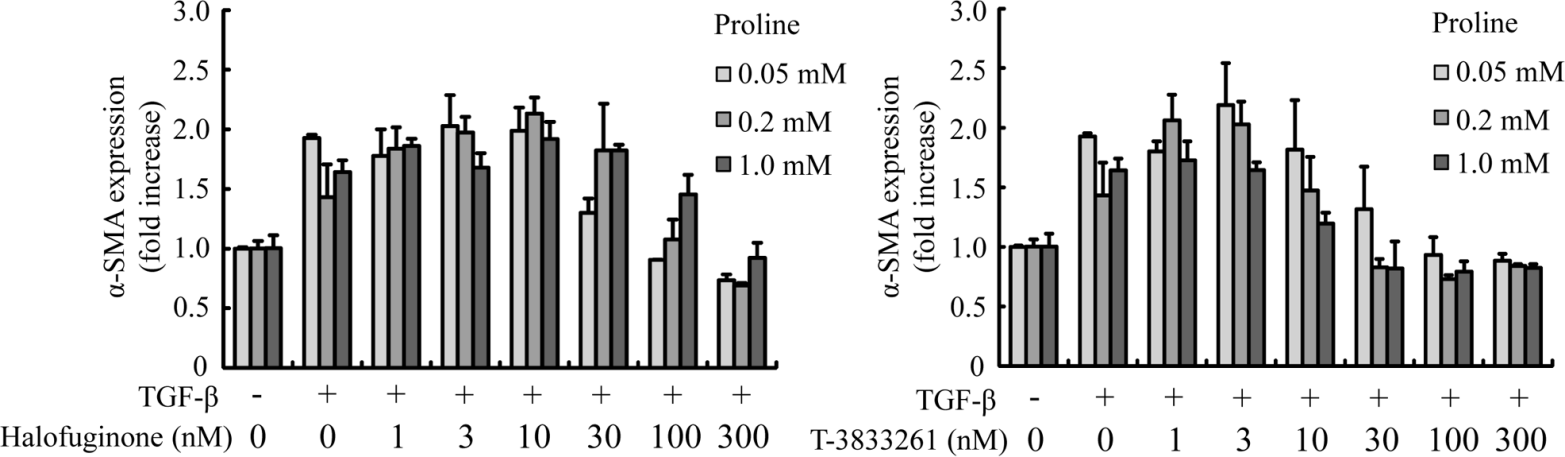
Effects of proline addition on PRS inhibitors (T-3833261 or Halofuginone)-induced reduction of α-SMA mRNA expression in skin fibroblasts. Skin fibroblasts were treated with or without T-3833261 or Halofuginone (1–300 nM) and/or proline (0.05, 0.2 and 1 mM) for 24 h. After incubation for 24 h, mRNA levels were measured by quantitative real-time RT-PCR. Gene expression levels (normalized to GAPDH) are expressed as the fold change of vehicle-treated control. Values are means ± SD (n = 3).

To verify the difference between the proline-competitive inhibitor and ATP-competitive inhibitor, we compared the effect of these inhibitors on a cellular fibrotic phenotype when exposed to proline addition. Concretely, we examined whether various concentrations of proline supplementation antagonized HF’s anti-fibrotic effect in skin fibroblast. The concentration of proline in human plasma is reported normally around 0.2–0.3 mM [21], and the proline content of normal tissues increases ca.5-fold in fibrotic tissues [14]. Therefore, we set the following assay condition; starting with 0.05 mM proline as a basal concentration in a FBM medium, proline concentration increases to 0.2 mM proline and 1.0 mM proline. HF-mediated inhibition of α-SMA gene expression was attenuated by adding proline to the medium, and this effect was proline concentration dependent manner (Fig 7). On the other hand, proline addition did not affect the activity of T-3833261 in a α-SMA expression.

## Discussion

HF has been shown to inhibit PRS and exert an anti-fibrotic effect *in vitro* and *in vivo* [8, 12, 22, 23], and it has been further shown to be efficacious in clinical use [10, 11]. In this study, we clearly showed that T-3833261 effectively inhibited TGF-β-induced fibrosis-related molecule expression *in vitro*. In a TGF-β-induced mouse ear fibrotic pharmacodynamic model that we established, the topical application of either HF or T-3833261 suppressed fibrotic gene expression. In view of existing structure–activity relationships, compounds with weak PRS inhibitory activity showed weak or no anti-fibrotic activity. Additionally, our ATP-competitive inhibitor T-3833261 showed almost no inhibitory activity against 27 kinases in a kinase panel experiment, similarly to inhibitors described in our previous report (IC_50_ >1000 nM) [16]. Taken together, we concluded that anti-fibrotic activity is highly associated with the PRS inhibitory activity observed by HF and T-3833261.

Since T-3833261 suppressed α-SMA protein expression (Fig 2) in accordance with a previous study on HF [8], we interested in the potential of T-3833261 to promote α-SMA protein degradation and reverse a differentiation process from fibroblast to myofibroblast. To confirm the promoting activity on α-SMA protein degradation, we tested the ability of T-3833261 and HF to induce dedifferentiation of myofibroblasts into fibroblasts, using TGF-β-induced differentiated skin myofibroblasts, which expressed high α-SMA levels. As a result, T-3833261 and HF promoted the reduction of α-SMA protein in a dose-dependent manner in TGF-β-induced differentiated myofibroblasts at 100 nM, and further reduced α-SMA levels to that of non-stimulated controls at 300 nM, (S1 Fig). A similar dedifferentiation effect was observed with 10-nitro-oleic acid treatment in lung myofibroblasts of an IPF patient that expressed high baseline α-SMA levels [24]. These findings indicate that both PRS inhibitors elicit activity capable of reversing established skin fibrosis in addition to prevention of fibrotic phenotype.

Concordant with the protein expression results, both T-3833261 and HF suppressed α-SMA and COL1A1 expression at the level of mRNA (Fig 4A). This result is consistent with suppression Smad3 signaling by PRS inhibition (Fig 3). The intensity of the Western blot band of Smad3 (p-Smad3) was normalized to β-actin and calculated by densitometry analysis (S2 Fig). We confirmed that T-3833261 and HF also induced the DDIT3 expression as an amino acid starvation response derived directly from PRS inhibition dose-dependently (Fig 4B). Furthermore, we confirmed that T-3833261 and HF increased gene expression of SMURF2 which has a role as a negative effector for TGF-β/Smad signaling under physiological conditions [25]. PRS inhibition-induced SMURF2 expression may be associated with suppression of Smad3 through disruption of Smad-complexes via mono-ubiquitination [26]. FGF2 induces the transition of myofibroblasts into fibroblasts via inhibition of Smad3 activity [27]. Kubo et al. [28] showed that FGF2 has potential effects on down-regulation of α-SMA expression *in vitro* [29]. In this study, T-3833261 and HF induced gene expression of FGF2 in a dose-dependent manner, i.e., PRS inhibitor has the ability to suppress TGF-β/Smad3 activity via upregulation of FGF2 expression. This effect further supported the transition effect of myofibroblasts into fibroblasts through downregulation of α-SMA expression by PRS inhibitor as described above. In this study, PRS inhibitors exerted these multiple mechanisms and showed anti-fibrotic effect through inhibition of both p-Smad3 and total Smad3.

This evidence is consistent with transcriptome data in human skin fibroblast with both types of PRS inhibitor treatment. HF altered expression levels of 8229 genes while T-3825026 (another ATP-competitive type PRS inhibitor) changed expressions of 8514 genes compared to vehicle-treated cells. The most of genes (7293 genes, 77%) of the total number of 9450 genes that commonly changed are overlapped between the two compounds (S3 Fig). These data indicated that HF and our ATP-competitive type PRS inhibitor are highly selective PRS inhibitors though these have different binding sites to PRS protein respectively. To explore an anti-fibrotic potency of PRS inhibitor-induced transcriptomes in an unbiased manner, we performed a comparison of gene expression between PRS inhibitor-induced common genes and fibroblast from scleroderma patients (GSE4385). Reverse overlapping expressions of genes that can be annotated by GO categories were mainly enriched in the regulation of extracellular matrix, extracellular matrix organization, extracellular structure organization and regulation of cell migration (S4 Fig). The strong relationship between PRS inhibitors and scleroderma was confirmed by gene signature analysis. However, further research is required for the detailed mechanism of the relationship between PRS inhibition and anti-fibrotic effect.

In order to evaluate the anti-fibrotic activity *in vivo*, the experimental conditions of the TGF-β-induced PD assay model was investigated. Chujo et al. [30] previously showed that serial injections of CTGF after TGF-β caused persistent fibrotic tissue formation in newborn mice. On the other hand, Yamaguchi et al. [31] reported that the injection of TGF-β (10 ng/mL, 100 μL) subcutaneously to the back of mice caused skin fibrosis after 1 week of injection. Based on this information, we evaluated some experimental conditions including injection site and administration term of TGF-β. As a result, we clarified that fibrotic gene expression depends on injection counts of TGF-β and that the fibrotic gene expression in the ear was a more sensitive site rather than dorsal skin against TGF-β stimulation (Fig 5). In the experimental condition, T-3833261 and HF reduced fibrotic genes expression in mouse skin treated with TGF-β (Fig 6). This suggests that the anti-fibrotic effect of both types of PRS inhibitors at topical treatment is due to its blockade of TGF-β activity *in vivo*. Based on these results we have a found promising lead compound that can be used in *in vivo* experiments. In this study, only one type of topical formulation was investigated for both HF and T-3833261. It is necessary to screen the optimal topical formulation for each active pharmaceutical ingredient to be selected for further development.

Next, we focused on the biochemical difference between HF and T-3833261 to identify the differentiation point of our ATP-competitive inhibitor *vs*. HF. Recent articles describing phenotypic resistance of protozoan cells toward HF treatment in malaria clearly indicate the potential issues in this class of drugs for use in cellular settings [13, 32]. After the treatment of HF, the substrate proline is accumulated in the cells and attenuates the cellular activity of HF. Along with our working hypothesis, we clarified that the T-3833261 did not show attenuated activity by proline addition (Fig 7). Kershenobich et al. [14] reported that a free proline content of cirrhotic liver was up to 4 fold increase compared to that of normal liver. This characteristic would provide our PRS inhibitor with a potential to work effectively in tissues where proline concentration increases with fibrosis progression. Details of the therapeutic effect *in vivo* need further investigation.

We found that ATP-competitive PRS inhibitor T-3833261 showed anti-fibrotic effect both *in vitro* and in *vivo*. One of the mechanisms of the anti-fibrotic action was inhibition of TGF-β/Smad3 signal. Our ATP-competitive PRS inhibitor approach would suggest one of the ways to inhibit fibrotic process effectively in progressive tissues with high proline concentration.

## Supporting information

S1 Fig Reverse fibrosis effect of T-3833261 or Halofuginone on α-SMA protein content in differentiated human skin myofibroblast. To differentiate myofibroblast, skin fibroblasts were stimulated with TGF-β (1 ng/mL) for 48 h. Then myofibloblast were treated with T-3833261 or Halofuginone (1–300 nM) without TGF-β. After incubation for 48 h, α-SMA protein levels were measured by ELISA. The expression levels are expressed as the percentage of vehicle-treated control. Values are mean ± SD (n = 4). #p<0.05 compared to vehicle-treated control, *p<0.05 compared to TGF-β-treated control. The experiment was repeated by using other fibroblast lots and similar results were obtained.
(TIF)

S2 Fig Quantification of Smad3 and p-Smad3 western blot data. The data is normalized to β-actin expression. The expression levels are expressed as the percentage of vehicle-treated control. Values are mean ± SE (n = 3). #p<0.05 compared to vehicle-treated control, *p<0.05 compared to TGF-β-treated control.
(TIF)

S3 Fig Venn diagram of T-3825026 and Halofuginone signature gene lists. PRS inhibitory signatures were defined as all genes showing >1.2 (or <1.2)-fold change (FDR < 0.05) after 24 h of addition of T-3825026 (another ATP-competitive type PRS inhibitor, PRS enzyme IC_50_:< 3.0×10^−9^, 300 nM) or Halofuginone (300 nM) in human skin fibroblast, respectively. The blue circle represents Halofuginone-induced differentially Expressed Gene (DEGs) at 300 nM in human skin fibroblast. The red circle represents T-3825026-induced DEGs at 300 nM in human skin fibroblast.
(TIF)

S4 Fig Venn diagram of signature gene lists in PRS inhibitor and fibroblast of scleroderma patient. The green circle represents both T-3825026 and Halofuginone-induced common DEGs at 300 nM in human skin fibroblast. The purple circle represents DEGs of fibroblast from scleroderma patient (GSSE4385, Sclerodermal fibroblasts forearm_vs_control) compared to that of healthy control. The 10 most highly correlated biological pathways overlapping changed genes of between PRS inhibitors and fibroblast of scleroderma patient.
(TIF)

S1 Table Taqman PCR primer sequences (mouse). Acta2, α-smooth muscle actin; Col1a1, type I collagen α; Col1a2, type II collagen α; Gapdh, glyceraldehyde 3-phosphate dehydrogenase.
(DOC)

S2 Table Body weight with treatment of PRS inhibitor on mouse. No significant difference was observed between the groups. Mean ± SE (n = 4–8).
(DOC)

## Abbreviations

α-SMA: α-smooth muscle actin
Col1a: type I collagen α
DDIT3: DNA damage inducible transcript 3
DEGs: differentially expressed genes
DMSO: dimethyl sulfoxide
ECM: extracellular matrix
FBM: fibroblast basal medium
FBS: fetal bovine serum
FGF2: fibroblast growth factor 2
GAPDH: glyceraldehyde 3-phosphate dehydrogenase
HF: halofuginone
mRSS: modified Rodnan skin score
PBS: phosphate-buffered saline
PD: pharmacodynamic
PRS: prolyl-tRNA synthetase
RT-PCR: reverse transcription-PCR
SMURF2: Smad specific E3 ubiquitin protein ligase 2
TGF-β: transforming growth factor-β
T-3833261: 2-Cyclopentyl-*N*-(3-fluoro-5-(1-methyl-1*H*-pyrazol-4-yl)benzyl)-3*H*-imidazo[4,5-*c*]pyridine-4-carboxamide
