# Supplementary figures and images for: Discovery and pharmacological characterization of a new class of prolyl-tRNA synthetase inhibitor for anti-fibrosis therapy
Source: PLoS One. 2017 Oct 24;12(10):e0186587. doi: 10.1371/journal.pone.0186587 (PMC5655428; doi:10.1371/journal.pone.0186587)

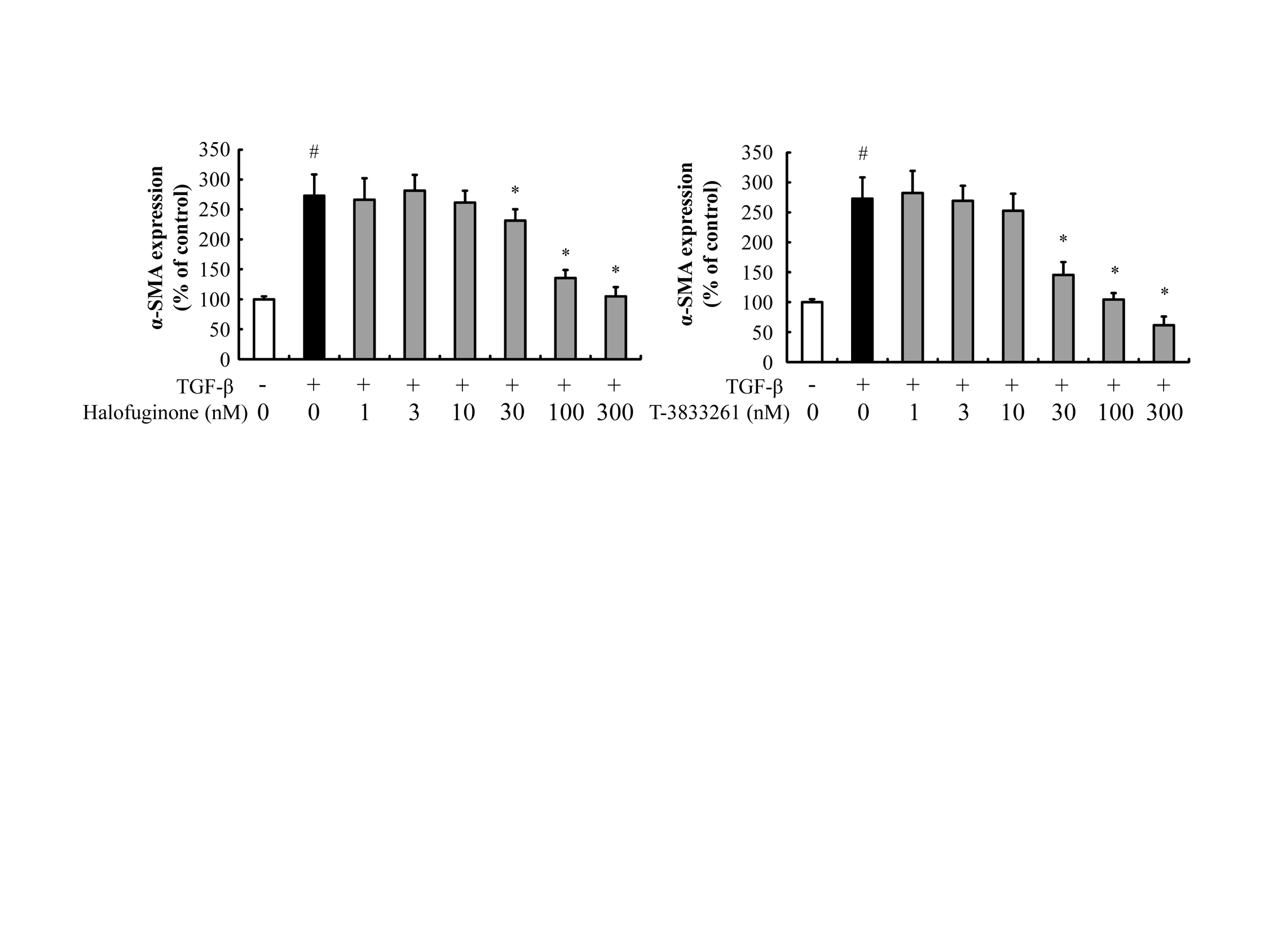

Supplement: S1 Fig — To differentiate myofibroblast, skin fibroblasts were stimulated with TGF-β (1 ng/mL) for 48 h. Then myofibloblast were treated with T-3833261 or Halofuginone (1–300 nM) without TGF-β. After incubation for 48 h, α-SMA protein levels were measured by ELISA. The expression levels are expressed as the percentage of vehicle-treated control. Values are mean ± SD (n = 4). #p<0.05 compared to vehicle-treated control, *p<0.05 compared to TGF-β-treated control. The experiment was repeated by using other fibroblast lots and similar results were obtained. (TIF) [file pone.0186587.s001.tif]

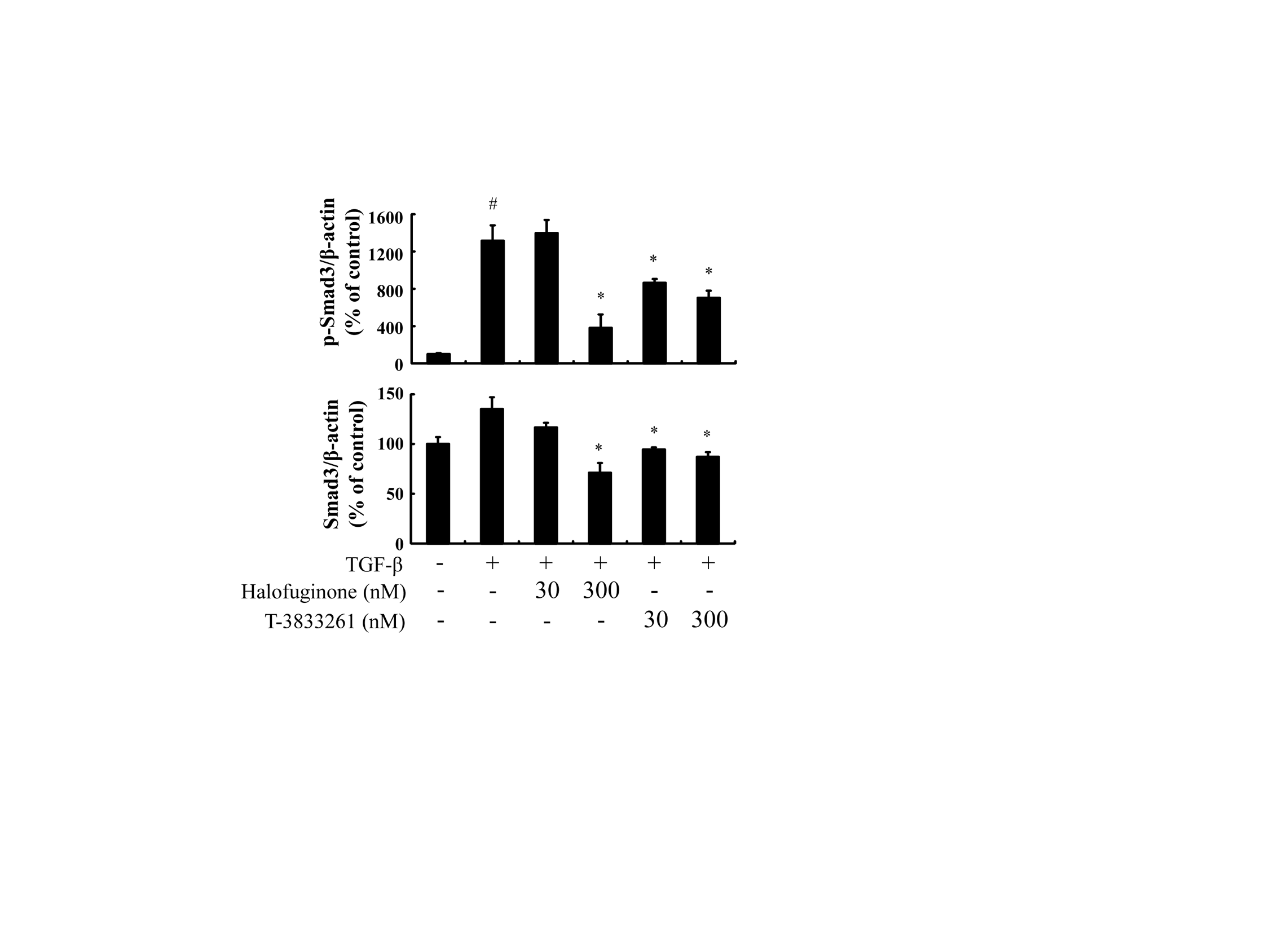

Supplement: S2 Fig — The data is normalized to β-actin expression. The expression levels are expressed as the percentage of vehicle-treated control. Values are mean ± SE (n = 3). #p<0.05 compared to vehicle-treated control, *p<0.05 compared to TGF-β-treated control. (TIF) [file pone.0186587.s002.tif]

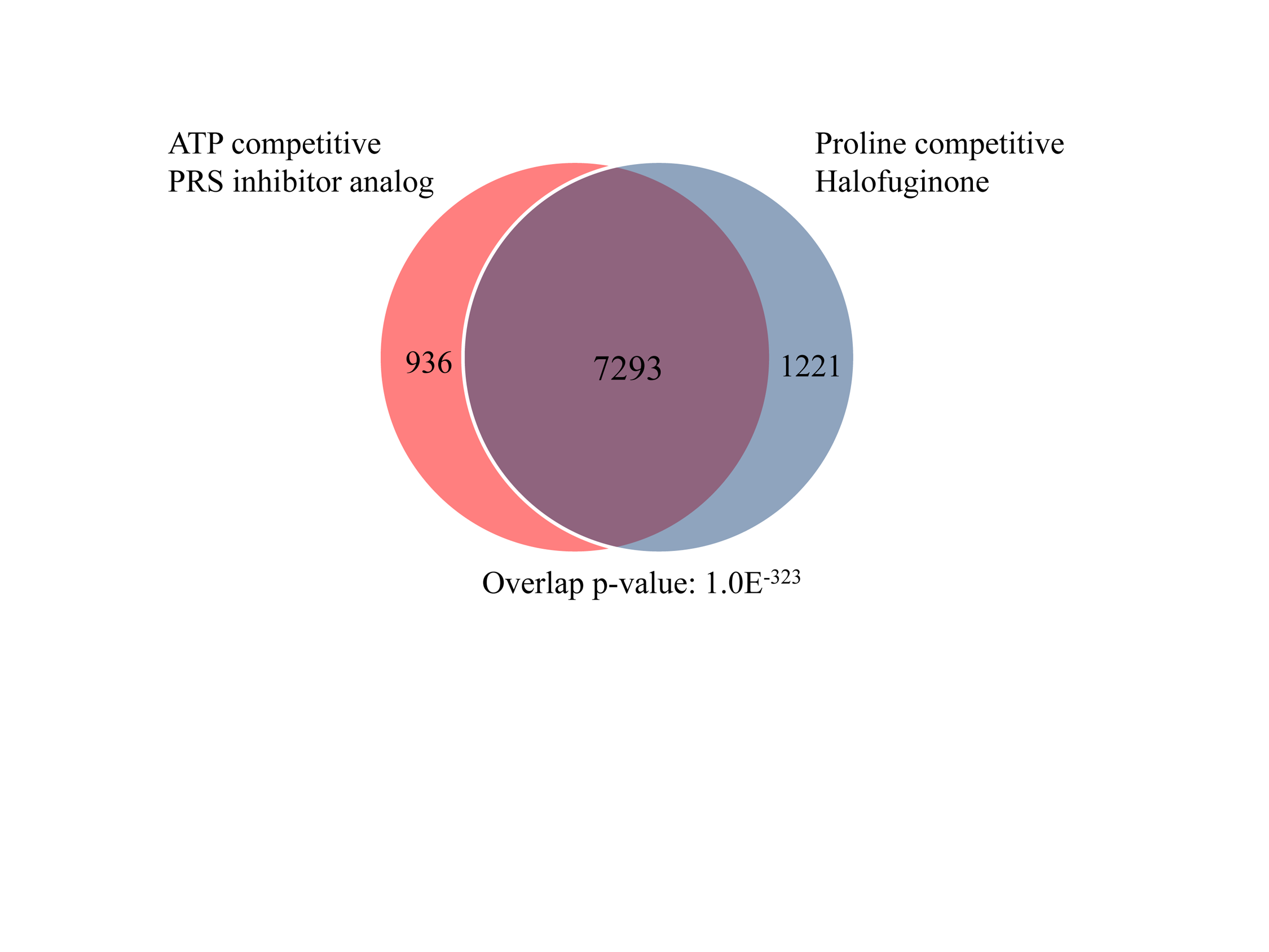

Supplement: S3 Fig — PRS inhibitory signatures were defined as all genes showing >1.2 (or <1.2)-fold change (FDR < 0.05) after 24 h of addition of T-3825026 (another ATP-competitive type PRS inhibitor, PRS enzyme IC50:< 3.0×10^−9^, 300 nM) or Halofuginone (300 nM) in human skin fibroblast, respectively. The blue circle represents Halofuginone-induced differentially Expressed Gene (DEGs) at 300 nM in human skin fibroblast. The red circle represents T-3825026-induced DEGs at 300 nM in human skin fibroblast. (TIF) [file pone.0186587.s003.tif]

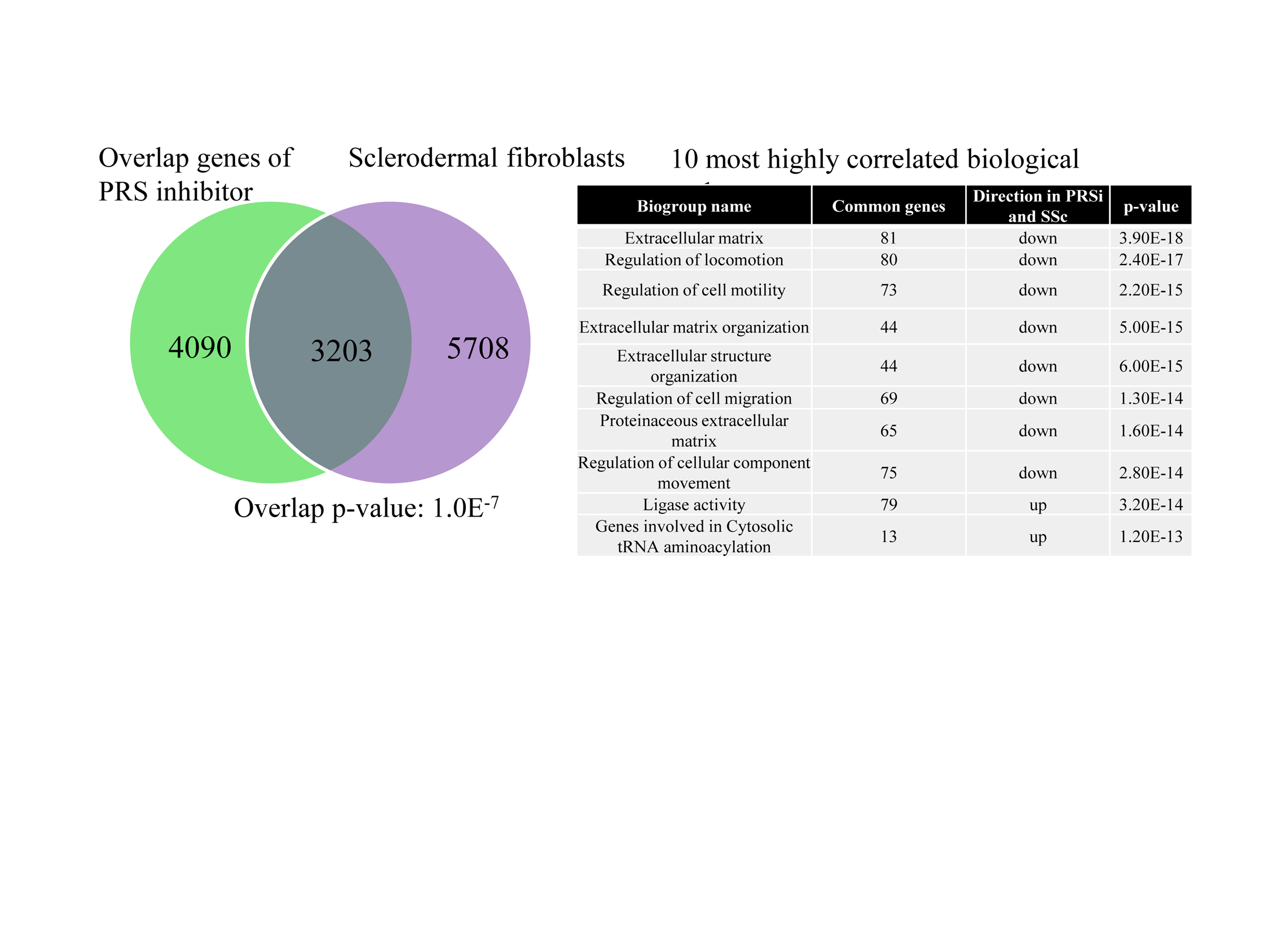

Supplement: S4 Fig — The green circle represents both T-3825026 and Halofuginone-induced common DEGs at 300 nM in human skin fibroblast. The purple circle represents DEGs of fibroblast from scleroderma patient (GSSE4385, Sclerodermal fibroblasts forearm_vs_control) compared to that of healthy control. The 10 most highly correlated biological pathways overlapping changed genes of between PRS inhibitors and fibroblast of scleroderma patient. (TIF) [file pone.0186587.s004.tif]
