# Supplementary material for: Discovery and pharmacological characterization of a new class of prolyl-tRNA synthetase inhibitor for anti-fibrosis therapy
Source: PLoS One. 2017 Oct 24;12(10):e0186587. doi: 10.1371/journal.pone.0186587 (PMC5655428; doi:10.1371/journal.pone.0186587)
Supplement: S1 Table — Acta2, α-smooth muscle actin; Col1a1, type I collagen α; Col1a2, type II collagen α; Gapdh, glyceraldehyde 3-phosphate dehydrogenase. (DOC) [file pone.0186587.s005.doc]

**Supplementary Table 1. Taqman PCR** primer sequences (mouse)

| Gene symbol | GenBank  Accession No. |  | Primer sequence (5'-3') |
| --- | --- | --- | --- |
| *Col1a1* | NM_007742 | Forward | GGACGGCTGCACGAGTCA |
|  |  | Reverse | CAGGCGGGAGGTCTTGGT |
|  |  | Probe | TTCGATGACTGTCTTGCCCCAAGTTCCG |
| *Col1a2* | NM_007743 | Forward | CAATGGTGGCAGCCAGTTTG |
|  |  | Reverse | TGTTCTGAGAAGCACGGTTGG |
|  |  | Probe | TGAGTTGCCATTTCCTTGGAGGACACCC |
| *Acta2* | NM_007392 | Forward | CGCTGAAGTATCCGATAGAACAC |
|  |  | Reverse | CGAAGCTCGTTATAGAAAGAGTGG |
|  |  | Probe | CATCATCACCAACTGGGACGACATGGAA |
| *Gapdh* | NM_008084 | Forward | CCAGCCTCGTCCCGTAGA |
|  |  | Reverse | TCTCCACTTTGCCACTGCA |
|  |  | Probe | CGGCCAAATCCGTTCACACCGACC |

Acta2, α-smooth muscle actin; Col1a1, type I collagen α; Col1a2, type II collagen α; Gapdh, glyceraldehyde 3-phosphate dehydrogenase
