# Supplementary material for: Discovery and pharmacological characterization of a new class of prolyl-tRNA synthetase inhibitor for anti-fibrosis therapy
Source: PLoS One. 2017 Oct 24;12(10):e0186587. doi: 10.1371/journal.pone.0186587 (PMC5655428; doi:10.1371/journal.pone.0186587)
Supplement: S2 Table — No significant difference was observed between the groups. Mean ± SE (n = 4–8). (DOC) [file pone.0186587.s006.doc]

**Supplemental Table 2. Body weight with treatment of PRS inhibitor on mouse**

|  | **Normal** | **Controls** | **0.01%**  **T-3833261** | **0.03%**  **T-3833261** | **0.1%**  **T-3833261** | **0.01%**  **Halofuginone** |
| --- | --- | --- | --- | --- | --- | --- |
| **Body weight day 0 (g)** | 23.1±0.8 | 22.2±0.5 | 22.9±0.8 | 23.1±0.6 | 22.4±0.4 | 22.5±0.3 |
| **Body weight day 7 (g)** | 23.1±0.7 | 23.0±0.5 | 23.8±0.5 | 23.4±0.4 | 22.2±0.5 | 23.7±0.3 |

No significant difference was observed between the groups. Mean ± SE (n = 4 - 8)
